# The genome sequence of the roach, *Rutilus rutilus* (Linnaeus, 1758) (Cypriniformes: Leuciscidae)

**DOI:** 10.12688/wellcomeopenres.25081.1

**Published:** 2025-11-06

**Authors:** Bernd Hänfling, Richard Pitman, Andy D. Nunn, Wellcome Sanger Institute Tree of Life Management, Samples and Laboratory team, Wellcome Sanger Institute Scientific Operations: Sequencing Operations, Wellcome Sanger Institute Tree of Life Core Informatics team, Tree of Life Core Informatics collective, Darwin Tree of Life Consortium

**Affiliations:** 1 Institute for Biodiversity and Freshwater Conservation, University of the Highlands and Islands, Inverness, Scotland, UK; 2 Environment Agency, Calverton, England, UK; 3 Hull International Fisheries Institute, School of Environmental and Life Sciences, University of Hull, Hull, England, UK

**Keywords:** Rutilus rutilus; Roach; genome sequence; chromosomal; Cypriniformes

## Abstract

We present a genome assembly from an individual *Rutilus rutilus* (Roach; Chordata; Actinopteri; Cypriniformes; Leuciscidae). The genome sequence has a total length of 1 100.18 megabases. Most of the assembly (99.56%) is scaffolded into 25 chromosomal pseudomolecules. The mitochondrial genome has also been assembled, with a length of 16.61 kilobases. Gene annotation of this assembly on Ensembl identified 25 358 protein-coding genes. This assembly was generated as part of the Darwin Tree of Life project, which produces reference genomes for eukaryotic species found in Britain and Ireland.

## Species taxonomy

Eukaryota; Opisthokonta; Metazoa; Eumetazoa; Bilateria; Deuterostomia; Chordata; Craniata; Vertebrata; Gnathostomata; Teleostomi; Euteleostomi; Actinopterygii; Actinopteri; Neopterygii; Teleostei; Osteoglossocephalai; Clupeocephala; Otomorpha; Ostariophysi; Otophysi; Cypriniphysae; Cypriniformes; Cyprinoidei; Leuciscidae; Leuciscinae; *Rutilus*; *Rutilus rutilus* (Linnaeus, 1758) (NCBI:txid48668)

## Background

The roach *Rutilus rutilus* (L.) is a freshwater teleost found throughout most of Europe and parts of western Asia, ranging from the British Isles in the west to the Ural Mountains and Caspian Basin in the east ( Kottelat & Freyhof, 2007). It is a relatively small species (usually <25 cm in length) that inhabits a variety of ecosystem types, including rivers, lakes, ponds and canals, as well as brackish waters in the Baltic Sea ( Kottelat & Freyhof, 2007). Roach typically become sexually mature at 2 to 4 years of age, with a maximum life span of less than 10 years in most populations ( Maitland, 2004).

Roach occupy a broad ecological niche, encompassing a diverse range of lotic and lentic environments, but are usually most abundant in slow-flowing rivers and productive still waters ( Maitland, 2004; Persson *et al.*, 1991). They are also relatively tolerant of poor water quality and physical habitat degradation ( Jeppesen *et al.*, 2000; Nunn *et al.*, 2025; Oberdorff *et al.*, 2002). Roach are omnivorous and adaptable foragers, consuming a diet that includes zooplankton, benthic invertebrates, plant material and detritus ( Hayden *et al.*, 2014; Nunn *et al.*, 2020). The lack of strict environmental and dietary requirements gives roach a competitive advantage over more sensitive and specialised species, meaning that it often dominates fish assemblages in eutrophic or heavily modified water bodies ( Jeppesen *et al.*, 2000; Nunn *et al.*, 2025; Persson *et al.*, 1991). Furthermore, the species can play a significant role in freshwater ecosystems by influencing nutrient dynamics, plankton communities and through its importance as prey ( Brabrand *et al.*, 1990; Cryer *et al.*, 1986; Russell *et al.*, 2022).

Despite its ubiquity, the species exhibits significant genetic structuring across its range, often reflecting watershed boundaries and historical colonisation patterns ( Laroche *et al.*, 1999). Habitat fragmentation by anthropogenic barriers, such as dams, has further contributed to reduced gene flow, leading to localised genetic divergence ( Hänfling *et al.*, 2004). Additionally, hybridisation with closely related species, particularly in heavily modified habitats, and widespread stocking have added complexity to its genetic landscape ( Hayden *et al.*, 2010). A chromosomal genome for the species has not previously been published, but scaffold-level genome assemblies for this species are also available (GCA_021464565.1, GCA_021464575.1, GCA_029875115.1, GCA_036024515.1; submitted by CSIRO Applied Genomics Initiative, the University of Lisbon, the Max Planck Institute of Molecular Cell Biology and Genetics) (NCBI datasets, O’Leary *et al.*, 2024).

Currently, the roach is listed as “Least Concern” on the International Union for the Conservation of Nature (IUCN) Red List of Threatened Species, owing to its broad distribution and large, stable populations ( Ford, 2024). Indeed, the species is relatively tolerant of the majority of threats affecting fresh waters ( Ford, 2024), but is impacted locally by poor water quality and habitat fragmentation ( Baynes *et al.*, 2023; Hänfling *et al.*, 2004).

**Figure 1.**
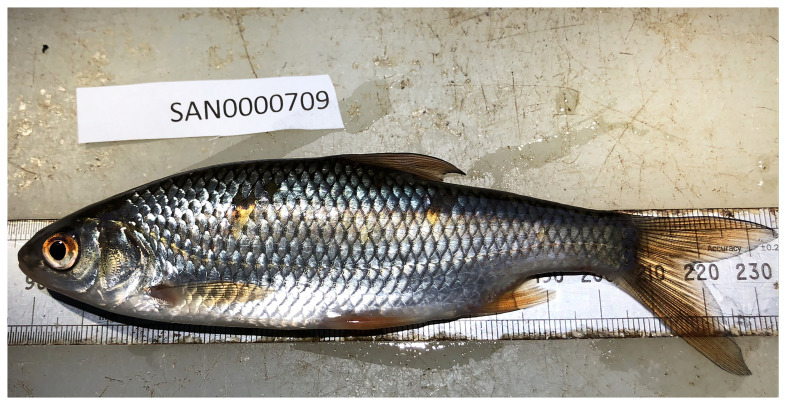
Photograph of the *Rutilus rutilus* (fRutRut2) specimen used for genome sequencing.

We present a chromosome-level genome sequence for *Rutilus rutilus*, produced using the Tree of Life pipeline from a specimen collected in Calverton Fish Farm, Nottingham, UK ( Figure 1). This assembly was generated as part of the Darwin Tree of Life Project, which aims to generate high-quality reference genomes for all named eukaryotic species in Britain and Ireland to support research, conservation, and the sustainable use of biodiversity ( Blaxter *et al.*, 2022).

## Methods

### Sample acquisition

One juvenile female *R. rutilus* (specimen ID SAN0000709, ToLID fRutRut2; Figure 1) was collected from the Environment Agency’s National Coarse Fish Rearing Unit in Calverton, Nottingham, UK (latitude 53.03, longitude –1.05) on 2020-08-25. The National Coarse Fish Rearing Unit at Calverton is funded solely through rod licence duty. The specimens were taken from the nursery pond by Richard Pitman (Environment Agency) using a seine net, and left to recover fully in fresh flowing, clean, borehole water for a week before any sampling commenced. The specimen was transported alive to the University of Hull where they were identified by Bernd Hänfling (University of Hull) and euthanised in a lethal dose of MS-222. Tissues dissection took place within 30 minutes of euthanasia, and the tissues were immediately shock-frozen in liquid nitrogen. Sample metadata were collected in line with the Darwin Tree of Life project standards described by Lawniczak *et al*. (2022).

### Nucleic acid extraction

Protocols for high molecular weight (HMW) DNA extraction developed at the Wellcome Sanger Institute (WSI) Tree of Life Core Laboratory are available on protocols.io ( Howard *et al.*, 2025). The fRutRut2 sample was weighed and triaged to determine the appropriate extraction protocol. Tissue from the spleen was homogenised by powermashing using a PowerMasher II tissue disruptor.

HMW DNA was extracted using the Automated MagAttract v2 protocol. DNA was sheared into an average fragment size of 12–20 kb following the Megaruptor®3 for LI PacBio protocol. Sheared DNA was purified by manual SPRI (solid-phase reversible immobilisation). The concentration of the sheared and purified DNA was assessed using a Nanodrop spectrophotometer and Qubit Fluorometer using the Qubit dsDNA High Sensitivity Assay kit. Fragment size distribution was evaluated by running the sample on the FemtoPulse system. For this sample, the final post-shearing DNA had a Qubit concentration of 39.8 ng/μL and a yield of 15 522.00 ng. The 260/280 spectrophotometric ratio was 1.83, and the 260/230 ratio was 2.02.

RNA was extracted from spleen tissue of fRutRut2 in the Tree of Life Laboratory at the WSI using the RNA Extraction: Automated MagMax™ *mir*Vana protocol. The RNA concentration was assessed using a Nanodrop spectrophotometer and a Qubit Fluorometer using the Qubit RNA Broad-Range Assay kit. Analysis of the integrity of the RNA was done using the Agilent RNA 6000 Pico Kit and Eukaryotic Total RNA assay.

### PacBio HiFi library preparation and sequencing

Library preparation and sequencing were performed at the WSI Scientific Operations core. Libraries were prepared using the SMRTbell Prep Kit 3.0 (Pacific Biosciences, California, USA), following the manufacturer’s instructions. The kit includes reagents for end repair/A-tailing, adapter ligation, post-ligation SMRTbell bead clean-up, and nuclease treatment. Size selection and clean-up were performed using diluted AMPure PB beads (Pacific Biosciences). DNA concentration was quantified using a Qubit Fluorometer v4.0 (ThermoFisher Scientific) and the Qubit 1X dsDNA HS assay kit. Final library fragment size was assessed with the Agilent Femto Pulse Automated Pulsed Field CE Instrument (Agilent Technologies) using the gDNA 55 kb BAC analysis kit.

The sample was sequenced using the Sequel IIe system (Pacific Biosciences, California, USA). The concentration of the library loaded onto the Sequel IIe was in the range 40–135 pM. The SMRT link software, a PacBio web-based end-to-end workflow manager, was used to set-up and monitor the run, and to perform primary and secondary analysis of the data upon completion.

### Hi-C

***Sample preparation and crosslinking***

The Hi-C sample was prepared from 20–50 mg of frozen gill tissue the fRutRut2 sample using the Arima-HiC v2 kit (Arima Genomics). Following the manufacturer’s instructions, tissue was fixed and DNA crosslinked using TC buffer to a final formaldehyde concentration of 2%. The tissue was homogenised using the Diagnocine Power Masher-II. Crosslinked DNA was digested with a restriction enzyme master mix, biotinylated, and ligated. Clean-up was performed with SPRISelect beads before library preparation. DNA concentration was measured with the Qubit Fluorometer (Thermo Fisher Scientific) and Qubit HS Assay Kit. The biotinylation percentage was estimated using the Arima-HiC v2 QC beads.

***Hi-C library preparation and sequencing***

Biotinylated DNA constructs were fragmented using a Covaris E220 sonicator and size selected to 400–600 bp using SPRISelect beads. DNA was enriched with Arima-HiC v2 kit Enrichment beads. End repair, A-tailing, and adapter ligation were carried out with the NEBNext Ultra II DNA Library Prep Kit (New England Biolabs), following a modified protocol where library preparation occurs while DNA remains bound to the Enrichment beads. Library amplification was performed using KAPA HiFi HotStart mix and a custom Unique Dual Index (UDI) barcode set (Integrated DNA Technologies). Depending on sample concentration and biotinylation percentage determined at the crosslinking stage, libraries were amplified with 10–16 PCR cycles. Post-PCR clean-up was performed with SPRISelect beads. Libraries were quantified using the AccuClear Ultra High Sensitivity dsDNA Standards Assay Kit (Biotium) and a FLUOstar Omega plate reader (BMG Labtech).

Prior to sequencing, libraries were normalised to 10 ng/μL. Normalised libraries were quantified again and equimolar and/or weighted 2.8 nM pools were created. Pool concentrations were checked using the Agilent 4200 TapeStation (Agilent) with High Sensitivity D500 reagents before sequencing. Sequencing was performed using paired-end 150 bp reads on the Illumina NovaSeq 6000.

### RNA library preparation and sequencing

Libraries were prepared using the NEBNext ^®^ Ultra™ II Directional RNA Library Prep Kit for Illumina (New England Biolabs), following the manufacturer’s instructions. Poly(A) mRNA in the total RNA solution was isolated using oligo(dT) beads, converted to cDNA, and uniquely indexed; 14 PCR cycles were performed. Libraries were size-selected to produce fragments between 100–300 bp. Libraries were quantified, normalised, pooled to a final concentration of 2.8 nM, and diluted to 150 pM for loading. Sequencing was carried out on the Illumina NovaSeq 6000 to generate 150-bp paired-end reads.

### Genome assembly

Prior to assembly of the PacBio HiFi reads, a database of *k*-mer counts ( *k* = 31) was generated from the filtered reads using FastK. GenomeScope2 ( Ranallo-Benavidez *et al.*, 2020) was used to analyse the *k*-mer frequency distributions, providing estimates of genome size, heterozygosity, and repeat content.

The HiFi reads were assembled using Hifiasm ( Cheng *et al.*, 2021) with the --primary option. Haplotypic duplications were identified and removed using purge_dups ( Guan *et al.*, 2020). The Hi-C reads ( Rao *et al.*, 2014) were mapped to the primary contigs using bwa-mem2 ( Vasimuddin *et al.*, 2019), and the contigs were scaffolded in YaHS ( Zhou *et al.*, 2023) with the --break option for handling potential misassemblies. The scaffolded assemblies were evaluated using Gfastats ( Formenti *et al.*, 2022), BUSCO ( Manni *et al.*, 2021) and MERQURY.FK ( Rhie *et al.*, 2020).

The mitochondrial genome was assembled using MitoHiFi ( Uliano-Silva *et al.*, 2023), which runs MitoFinder ( Allio *et al.*, 2020) and uses these annotations to select the final mitochondrial contig and to ensure the general quality of the sequence.

### Assembly curation

The assembly was decontaminated using the Assembly Screen for Cobionts and Contaminants ( ASCC) pipeline. TreeVal was used to generate the flat files and maps for use in curation. Manual curation was conducted primarily in PretextView and HiGlass ( Kerpedjiev *et al.*, 2018). Scaffolds were visually inspected and corrected as described by Howe *et al*. (2021). Manual corrections included 38 breaks, 47 joins, and removal of 23 haplotypic duplications. The curation process is documented at https://gitlab.com/wtsi-grit/rapid-curation. PretextSnapshot was used to generate a Hi-C contact map of the final assembly.

### Assembly quality assessment

The Merqury.FK tool ( Rhie *et al.*, 2020) was run in a Singularity container ( Kurtzer *et al.*, 2017) to evaluate *k*-mer completeness and assembly quality for the primary and alternate haplotypes using the *k*-mer databases ( *k* = 31) computed prior to genome assembly. The analysis outputs included assembly QV scores and completeness statistics.

The genome was analysed using the BlobToolKit pipeline, a Nextflow implementation of the earlier Snakemake version ( Challis *et al.*, 2020). The pipeline aligns PacBio reads using minimap2 ( Li, 2018) and SAMtools ( Danecek *et al.*, 2021) to generate coverage tracks. It runs BUSCO ( Manni *et al.*, 2021) using lineages identified from the NCBI Taxonomy ( Schoch *et al.*, 2020). For the three domain-level lineages, BUSCO genes are aligned to the UniProt Reference Proteomes database ( Bateman *et al.*, 2023) using DIAMOND blastp ( Buchfink *et al.*, 2021). The genome is divided into chunks based on the density of BUSCO genes from the closest taxonomic lineage, and each chunk is aligned to the UniProt Reference Proteomes database with DIAMOND blastx. Sequences without hits are chunked using seqtk and aligned to the NT database with blastn ( Altschul *et al.*, 1990). The BlobToolKit suite consolidates all outputs into a blobdir for visualisation. The BlobToolKit pipeline was developed using nf-core tooling ( Ewels *et al.*, 2020) and MultiQC ( Ewels *et al.*, 2016), with containerisation through Docker ( Merkel, 2014) and Singularity ( Kurtzer *et al.*, 2017).

## Genome sequence report

### Sequence data

**Figure 2.**
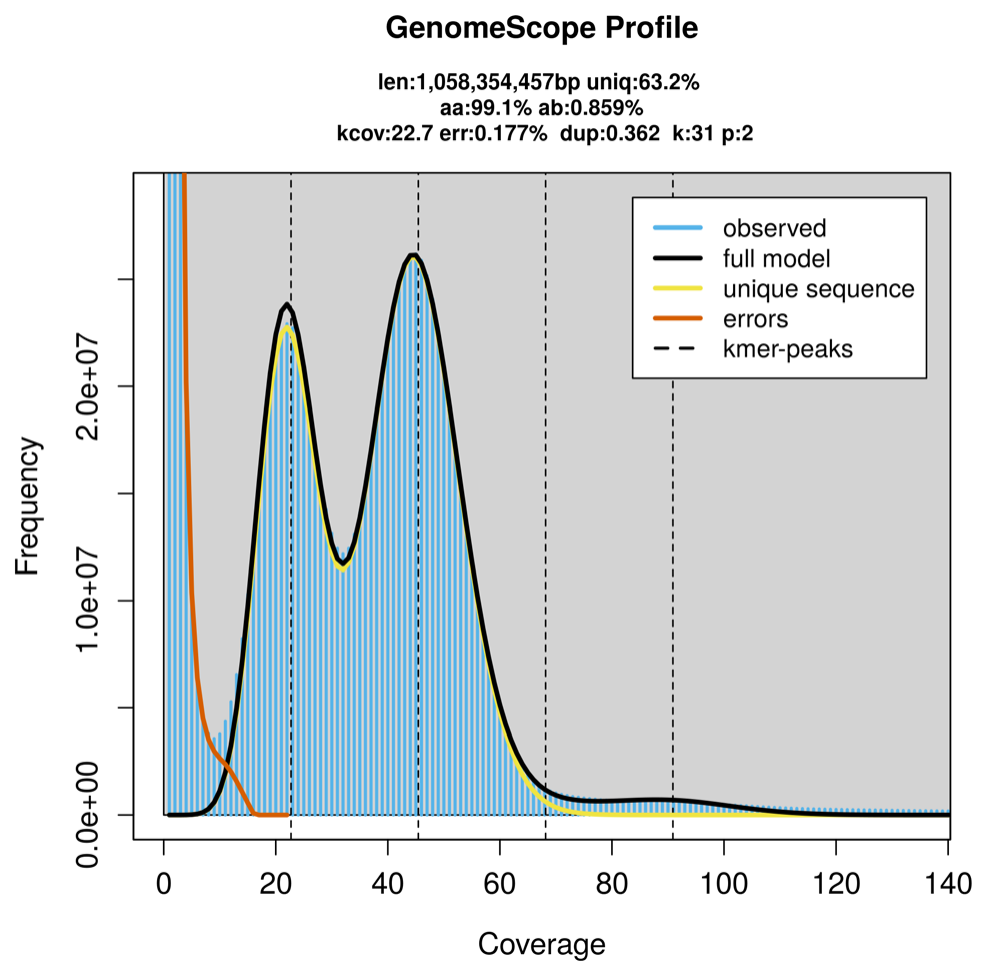
Frequency distribution of *k*-mers generated using GenomeScope2. The plot shows observed and modelled *k*-mer spectra, providing estimates of genome size, heterozygosity, and repeat content based on unassembled sequencing reads.

PacBio sequencing of the *Rutilus rutilus* specimen generated 50.93 Gb (gigabases) from 4.24 million reads, which were used to assemble the genome. GenomeScope2.0 analysis estimated the haploid genome size at 1 058.35 Mb, with a heterozygosity of 0.86% and repeat content of 36.96% ( Figure 2). These estimates guided expectations for the assembly. Based on the estimated genome size, the sequencing data provided approximately 45× coverage. Hi-C sequencing produced 144.92 Gb from 959.71 million reads, which were used to scaffold the assembly. RNA sequencing data were also generated and are available in public sequence repositories. Table 1 summarises the specimen and sequencing details.

**Table 1.** Specimen and sequencing data for BioProject PRJEB61621.

Platform	PacBio HiFi	Hi-C	RNA-seq
**ToLID**	fRutRut2	fRutRut2	fRutRut2
**Specimen ID**	SAN0000709	SAN0000709	SAN0000709
**BioSample (source individual)**	SAMEA11296543	SAMEA11296543	SAMEA11296543
**BioSample (tissue)**	SAMEA11296642	SAMEA11296639	SAMEA11296642
**Tissue**	spleen	gill animal	spleen
**Instrument**	Sequel IIe	Illumina NovaSeq 6000	Illumina NovaSeq 6000
**Run accessions**	ERR11279093; ERR11279094	ERR11271536	ERR12245568
**Read count total**	4.24 million	959.71 million	69.86 million
**Base count total**	50.93 Gb	144.92 Gb	10.55 Gb

### Assembly statistics

The primary haplotype was assembled, and contigs corresponding to an alternate haplotype were also deposited in INSDC databases. The final assembly has a total length of 1 100.18 Mb in 84 scaffolds, with 471 gaps, and a scaffold N50 of 43.94 Mb ( Table 2).

**Table 2.** Genome assembly statistics.

**Assembly name**	fRutRut2.1
**Assembly accession**	GCA_951802725.1
**Alternate haplotype accession**	GCA_951802655.1
**Assembly level**	chromosome
**Span (Mb)**	1 100.18
**Number of chromosomes**	25
**Number of contigs**	555
**Contig N50**	3.93 Mb
**Number of scaffolds**	84
**Scaffold N50**	43.94 Mb
**Organelles**	Mitochondrion: 16.61 kb

**Figure 3.**
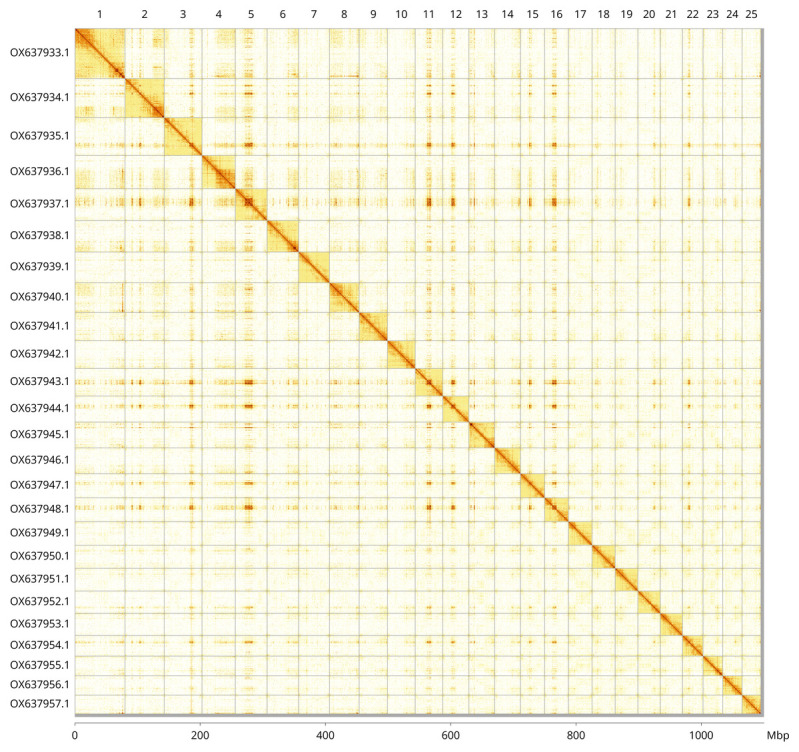
Hi-C contact map of the *Rutilus rutilus* genome assembly. Assembled chromosomes are shown in order of size and labelled along the axes, with a megabase scale shown below. The plot was generated using PretextSnapshot.

Most of the assembly sequence (99.56%) was assigned to 25 chromosomal-level scaffolds. These chromosome-level scaffolds, confirmed by Hi-C data, are named according to size ( Figure 3; Table 3). During curation, we observed that there is a large heterozygous inversion on Chromosome 9 between 4.9 Mb and 15.9 Mb.

**Table 3.** Chromosomal pseudomolecules in the primary genome assembly of *Rutilus rutilus* fRutRut2.

INSDC accession	Molecule	Length (Mb)	GC%
OX637933.1	1	80.29	39
OX637934.1	2	62.44	38.50
OX637935.1	3	60.22	39
OX637936.1	4	53.34	39.50
OX637937.1	5	50.61	39
OX637938.1	6	50.38	39
OX637939.1	7	49.08	38.50
OX637940.1	8	47.67	39
OX637941.1	9	45.04	39.50
OX637942.1	10	44.28	39
OX637943.1	11	43.94	38.50
OX637944.1	12	41.90	38.50
OX637945.1	13	41.12	39
OX637946.1	14	41	39
OX637947.1	15	38.72	38.50
OX637948.1	16	38.06	38.50
OX637949.1	17	37.65	38.50
OX637950.1	18	36.98	38.50
OX637951.1	19	36.08	38.50
OX637952.1	20	35.86	38.50
OX637953.1	21	35.34	38.50
OX637954.1	22	32.92	39
OX637955.1	23	31.30	39
OX637956.1	24	30.86	38.50
OX637957.1	25	30.22	38.50

The mitochondrial genome was also assembled. This sequence is included as a contig in the multifasta file of the genome submission and as a standalone record.

**Figure 4.**
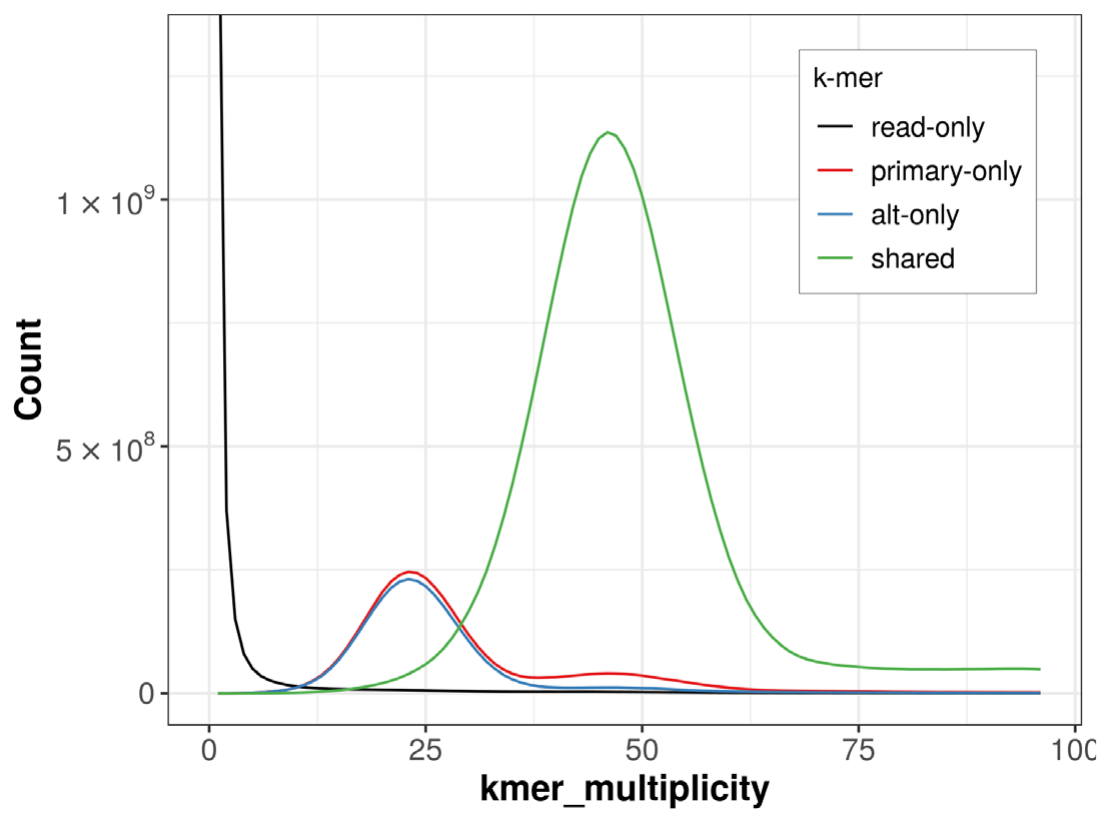
Evaluation of *k*-mer completeness using MerquryFK. This plot illustrates the recovery of *k*-mers from the original read data in the final assemblies. The horizontal axis represents *k*-mer multiplicity, and the vertical axis shows the number of *k*-mers. The black curve represents *k*-mers that appear in the reads but are not assembled. The green curve corresponds to *k*-mers shared by both haplotypes, and the red and blue curves show *k*-mers found only in one of the haplotypes.

The combined primary and alternate assemblies achieve an estimated QV of 59.0. The *k*-mer completeness is 82.43% for the primary assembly, 79.95% for the alternate haplotype, and 98.61% for the combined assemblies ( Figure 4).

**Figure 5.**
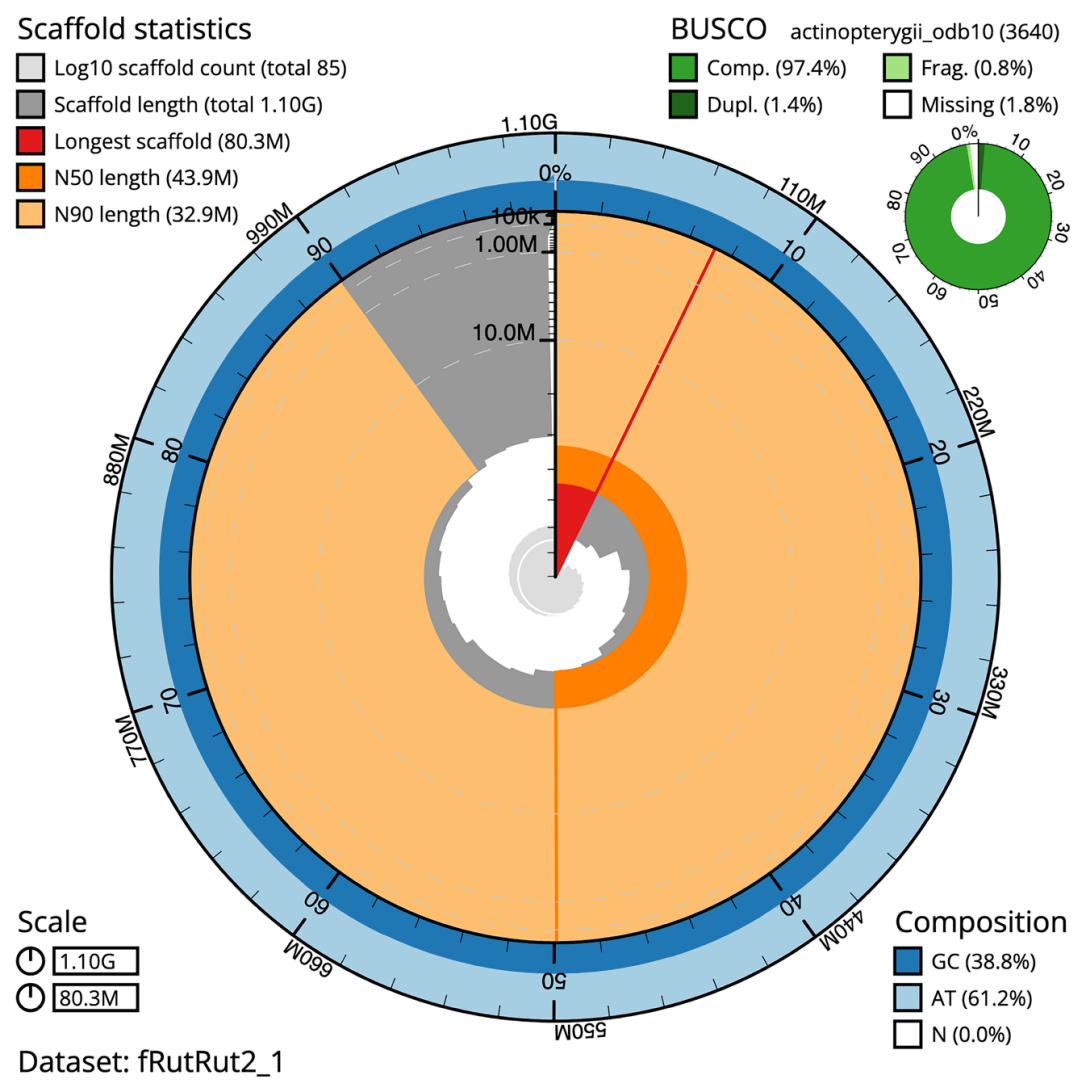
Assembly metrics for fRutRut2.1. The BlobToolKit snail plot provides an overview of assembly metrics and BUSCO gene completeness. The circumference represents the length of the whole genome sequence, and the main plot is divided into 1 000 bins around the circumference. The outermost blue tracks display the distribution of GC, AT, and N percentages across the bins. Scaffolds are arranged clockwise from longest to shortest and are depicted in dark grey. The longest scaffold is indicated by the red arc, and the deeper orange and pale orange arcs represent the N50 and N90 lengths. A light grey spiral at the centre shows the cumulative scaffold count on a logarithmic scale. A summary of complete, fragmented, duplicated, and missing BUSCO genes in the set is presented at the top right. An interactive version of this figure can be accessed on the BlobToolKit viewer.

**Figure 6.**
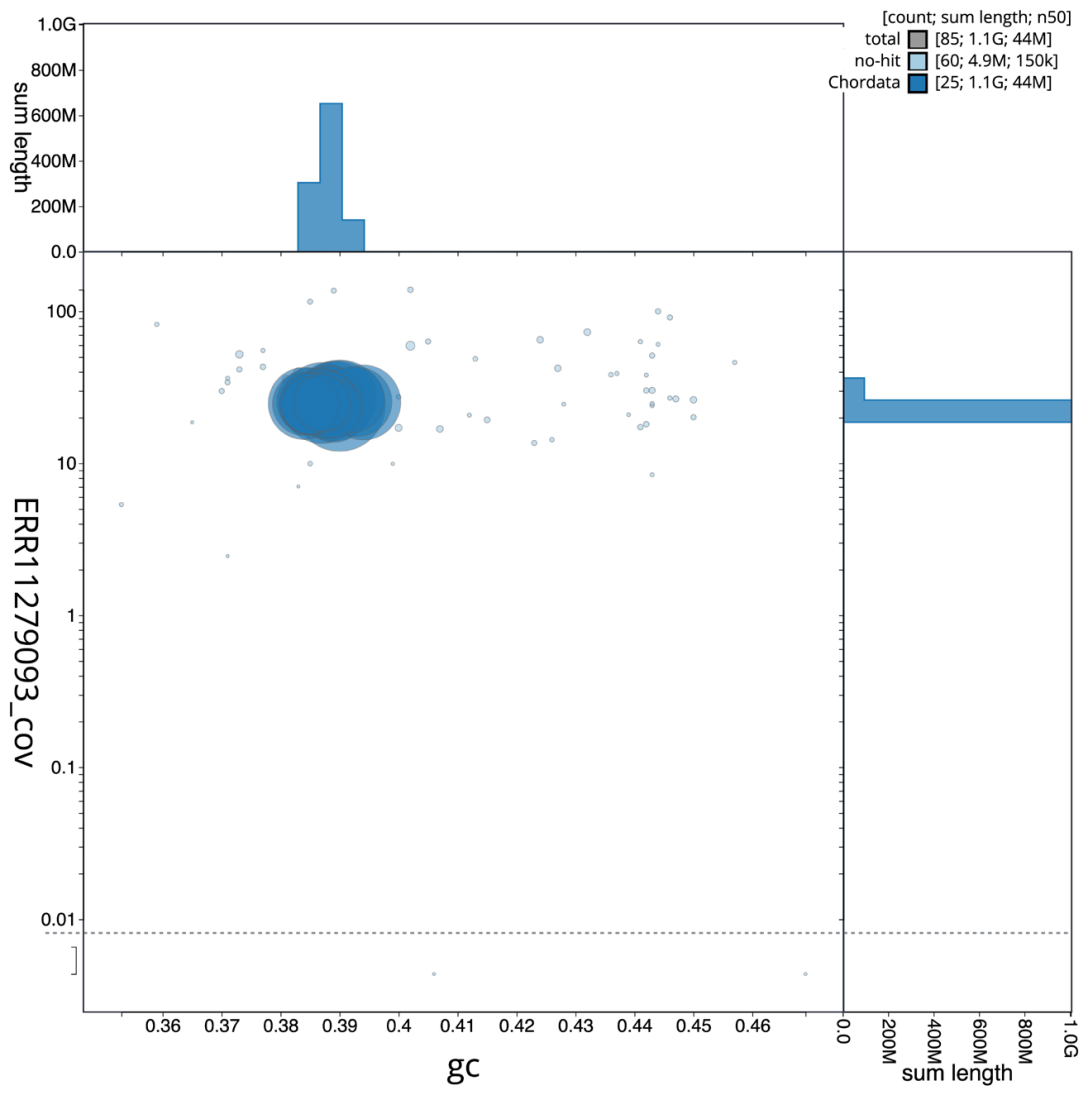
BlobToolKit GC-coverage plot for fRutRut2.1. Blob plot showing sequence coverage (vertical axis) and GC content (horizontal axis). The circles represent scaffolds, with the size proportional to scaffold length and the colour representing phylum membership. The histograms along the axes display the total length of sequences distributed across different levels of coverage and GC content. An interactive version of this figure is available on the BlobToolKit viewer.

BUSCO v. 5.3.2 analysis using the actinopterygii_odb10 reference set ( *n* = 3 640) identified 97.4 % of the expected gene set (single = 96.0%, duplicated = 1.4%). The snail plot in Figure 5 summarises the scaffold length distribution and other assembly statistics for the primary assembly. The blob plot in Figure 6 shows the distribution of scaffolds by GC proportion and coverage.

Table 4 lists the assembly metric benchmarks adapted from Rhie *et al.* (2021) and the Earth BioGenome Project Report on Assembly Standards September 2024. The EBP metric, calculated for the primary assembly, is **6.C.Q59**, meeting the recommended reference standard.

**Table 4.** Earth Biogenome Project summary metrics for the *Rutilus rutilus* assembly.

Measure	Value	Benchmark
EBP summary (primary)	6.C.Q59	6.C.Q40
Contig N50 length	3.93 Mb	≥ 1 Mb
Scaffold N50 length	43.94 Mb	= chromosome N50
Consensus quality (QV)	Primary: 59.0; alternate: 59.1; combined: 59.0	≥ 40
*k*-mer completeness	Primary: 82.43%; alternate: 79.95%; combined: 98.61%	≥ 95%
BUSCO	C:97.4%[S:96.0%;D:1.4%];F:0.8%;M:1.8%;n:3 640	S > 90%; D < 5%
Percentage of assembly assigned to chromosomes	99.56%	≥ 90%

## Genome annotation report

The *Rutilus rutilus* genome assembly (GCA_951802725.1) was annotated by Ensembl at the European Bioinformatics Institute (EBI) using the Ensembl genebuild method. This annotation includes 89 683 transcribed mRNAs from 25 358 protein-coding and 16 409 non-coding genes. The average transcript length is 23 380.94 bp, with an average of 2.14 coding transcripts per gene and 11.76 exons per transcript. For further information about the annotation, please refer to the annotation page on Ensembl.

### Wellcome Sanger Institute – Legal and Governance

The materials that have contributed to this genome note have been supplied by a Darwin Tree of Life Partner. The submission of materials by a Darwin Tree of Life Partner is subject to the **‘Darwin Tree of Life Project Sampling Code of Practice’**, which can be found in full on the Darwin Tree of Life website. By agreeing with and signing up to the Sampling Code of Practice, the Darwin Tree of Life Partner agrees they will meet the legal and ethical requirements and standards set out within this document in respect of all samples acquired for, and supplied to, the Darwin Tree of Life Project. Further, the Wellcome Sanger Institute employs a process whereby due diligence is carried out proportionate to the nature of the materials themselves, and the circumstances under which they have been/are to be collected and provided for use. The purpose of this is to address and mitigate any potential legal and/or ethical implications of receipt and use of the materials as part of the research project, and to ensure that in doing so we align with best practice wherever possible. The overarching areas of consideration are:

- Ethical review of provenance and sourcing of the material
- Legality of collection, transfer and use (national and international)

Each transfer of samples is further undertaken according to a Research Collaboration Agreement or Material Transfer Agreement entered into by the Darwin Tree of Life Partner, Genome Research Limited (operating as the Wellcome Sanger Institute), and in some circumstances, other Darwin Tree of Life collaborators.

## Data Availability

European Nucleotide Archive: Rutilus rutilus (roach minnow). Accession number PRJEB61621. The genome sequence is released openly for reuse. The *Rutilus rutilus* genome sequencing initiative is part of the Darwin Tree of Life Project (PRJEB40665), the Sanger Institute Tree of Life Programme (PRJEB43745) and Vertebrate Genomes Project (PRJNA489243). All raw sequence data and the assembly have been deposited in INSDC databases. Raw data and assembly accession identifiers are reported in Table 1 and Table 2. Production code used in genome assembly at the WSI Tree of Life is available at https://github.com/sanger-tol. Table 5 lists software versions used in this study.

## Author information

Contributors are listed at the following links:

- Members of the Wellcome Sanger Institute Tree of Life Management, Samples and Laboratory team
- Members of Wellcome Sanger Institute Scientific Operations – Sequencing Operations
- Members of the Wellcome Sanger Institute Tree of Life Core Informatics team
- Members of the Tree of Life Core Informatics collective
- Members of the Darwin Tree of Life Consortium
