# Supplementary material for: The genome sequence of the roach, Rutilus rutilus (Linnaeus, 1758) (Cypriniformes: Leuciscidae)
Source: Wellcome Open Res. 2025 Nov 6;10:629. [Version 1] doi: 10.12688/wellcomeopenres.25081.1 (PMC12789864; doi:10.12688/wellcomeopenres.25081.1)
Supplement: Peer review history

# Reviewer report: Reviewer response for version 1

**DOI:** 10.21956/wellcomeopenres.27649.r143811

**Published:** 2026-03-12

**Authors:** Josephine R Paris

**Affiliations:** 1 Department of Biology, Colorado State University, Fort Collins, CO, USA

The background is well written and informative. The genome assembly meets the high standards of the Wellcome Sanger Institute Tree of Life. It's great to see an improvement on the previous scaffold-level assemblies, especially with such a high percentage of the assembly scaffolded into 25 chromosomes. Methods are well explained and clear. Data are available via appropriate data repositories.

Are sufficient details of methods and materials provided to allow replication by others?

Yes

Is the rationale for creating the dataset(s) clearly described?

Yes

Are the datasets clearly presented in a useable and accessible format?

Yes

Are the protocols appropriate and is the work technically sound?

Yes

Reviewer Expertise:

population genomics and genome assembly of non-model vertebrates

I confirm that I have read this submission and believe that I have an appropriate level of expertise to confirm that it is of an acceptable scientific standard.

# Reviewer report: Reviewer response for version 1

**DOI:** 10.21956/wellcomeopenres.27649.r143809

**Published:** 2026-01-09

**Authors:** Alejandro S. Mechaly

**Affiliations:** 1 Instituto de Investigaciones en Biodiversidad y Biotecnología (INBIOTEC-CONICET), Mar del Plata, Argentina

**This data note by Hänfling and collaborators presents a valuable genomic resource for** *Rutilus rutilus* **(roach), a widespread freshwater fish species distributed across Europe and parts of western Asia.** Although the species is not of major commercial importance and is currently classified as *Least Concern* by the IUCN, it plays an important ecological role in freshwater ecosystems and has been widely used as a biological model in environmental and ecotoxicological research, particularly in studies related to water quality, endocrine disruption, and other anthropogenic stressors.

 The study reports the first high-quality chromosome-level genome assembly for *R. rutilus*, generated using PacBio HiFi sequencing complemented with Hi-C scaffolding and RNA-seq data. The resulting assembly shows very good contiguity and completeness, with most of the genome anchored to chromosomal pseudomolecules and a high BUSCO recovery, in line with current standards for reference genomes.

 The availability of a well-curated reference genome is especially relevant for *R. rutilus*, given its frequent use in studies addressing pollution impacts, population structure, hybridisation, and ecological resilience in freshwater systems affected by human activities. This resource will be useful for future work in conservation genomics, ecotoxicology, and evolutionary ecology, and may also support applied research in freshwater ecosystem management.

 Overall, this study provides a solid genomic resource that will be of broad interest to the research community.

**Minor comment**

 While the ecological relevance of *Rutilus rutilus* is well established, the authors may consider briefly highlighting its role as a model species in environmental monitoring and ecotoxicological studies (e.g. water quality assessment, endocrine disruption, responses to anthropogenic stressors), as this would further emphasize the applied relevance of generating a chromosome-level reference genome for this species.

Are sufficient details of methods and materials provided to allow replication by others?

Yes

Is the rationale for creating the dataset(s) clearly described?

Yes

Are the datasets clearly presented in a useable and accessible format?

Yes

Are the protocols appropriate and is the work technically sound?

Yes

Reviewer Expertise:

Fish reproduction and neuroendocrinology

I confirm that I have read this submission and believe that I have an appropriate level of expertise to confirm that it is of an acceptable scientific standard.

# Reviewer report: Reviewer response for version 1

**DOI:** 10.21956/wellcomeopenres.27649.r139117

**Published:** 2025-11-25

**Authors:** Jayan Duminda M Senevirathna

**Affiliations:** 1 Uva Wellassa University, Badulla, Uva Province, Sri Lanka

The manuscript presents a high-quality draft genome assembly of *Rutilus rutilus* and is overall very well written, clearly structured, and scientifically sound. The authors provide a strong justification for sequencing this species and have successfully generated an assembly with robust metrics, including high BUSCO completeness, appropriate sequencing depth, and well-executed contamination filtering.

 The integration of assembly statistics, and annotation workflows are there however, incorporating comparative genomic insights can strengthens the manuscript and enhances its relevance for future evolutionary, ecological, and conservation studies.

 The methods are detailed enough to ensure reproducibility, and the results are presented with clarity. Figures and tables are well designed, particularly the assembly summary and blob plot, which effectively illustrate genome quality and taxonomic confidence. Data availability is clearly stated, making the work transparent and useful for the broader research community.

 Overall, this is a solid contribution to fish genomics, and I recommend acceptance with only minor revisions to further improve clarity (e.g., adding more detail on annotation parameter settings and a brief discussion on potential assembly limitations). With these minor adjustments, the manuscript will be very strong.

Are sufficient details of methods and materials provided to allow replication by others?

Yes

Is the rationale for creating the dataset(s) clearly described?

Yes

Are the datasets clearly presented in a useable and accessible format?

Yes

Are the protocols appropriate and is the work technically sound?

Yes

Reviewer Expertise:

Aquatic Biotechnology and Genetics

I confirm that I have read this submission and believe that I have an appropriate level of expertise to confirm that it is of an acceptable scientific standard.
